# Supplementary material for: Structural and dynamic mechanisms of CBF3-guided centromeric nucleosome formation
Source: Nat Commun. 2021 Mar 19;12:1763. doi: 10.1038/s41467-021-21985-9 (PMC7979930; doi:10.1038/s41467-021-21985-9)
Supplement: Supplementary file 1 — Supplementary Information [file 41467_2021_21985_MOESM1_ESM.pdf]

Supplementary Information

Structural and dynamic mechanisms of CBF3-guided centromeric nucleosome formation

Guan et al.

Supplementary Figs. 1-4

Supplementary Table 1

Supplementary Table 2

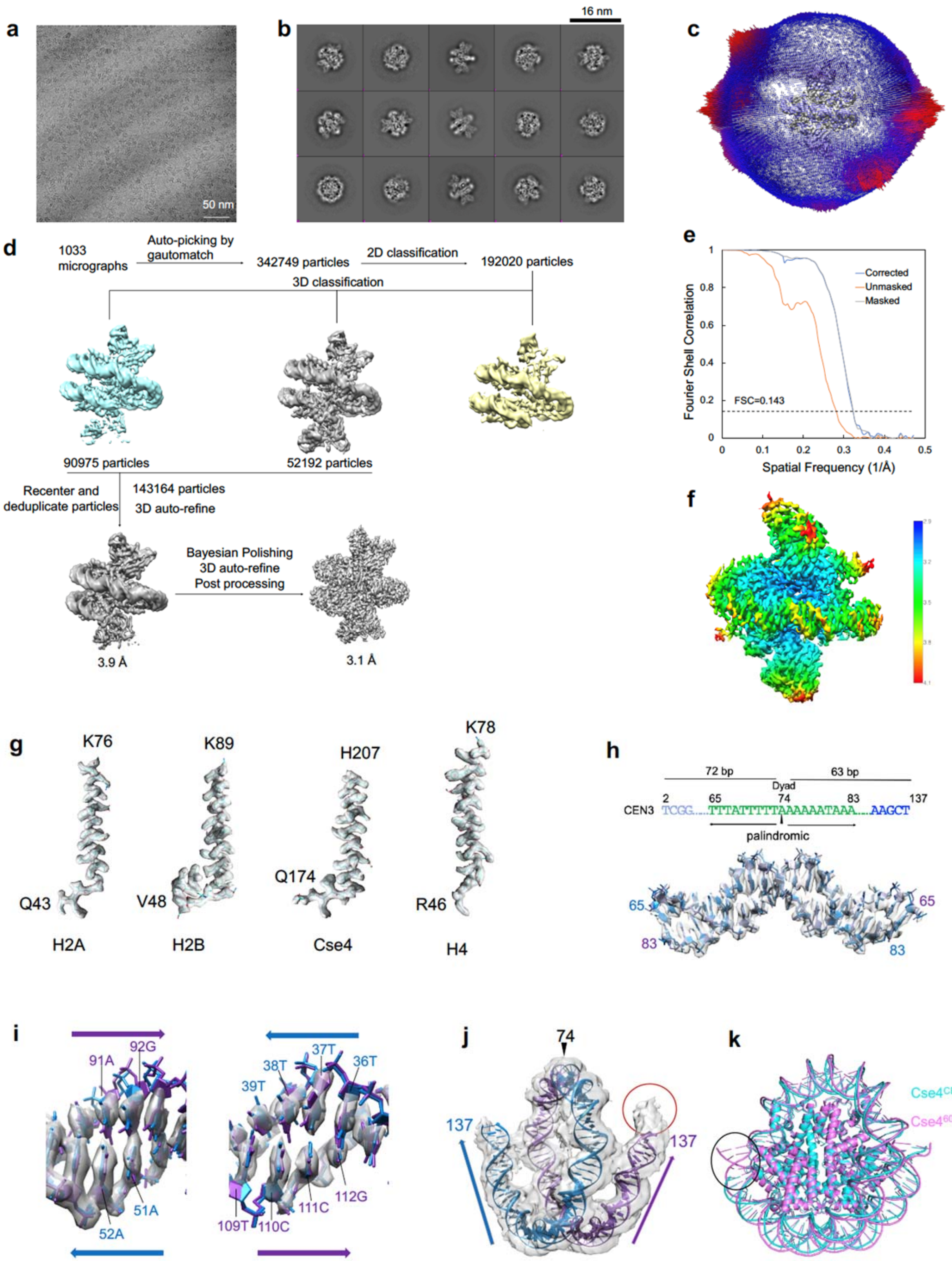
a
50 nm
b
16 nm
c
d
1033 micrographs
Auto-picking by gautomatch
342749 particles
2D classification
192020 particles
3D classification
90975 particles
52192 particles
Recenter and deduplicate particles
143164 particles
3D auto-refine
Bayesian Polishing
3D auto-refine
Post processing
3.9 Å
3.1 Å
e
Fourier Shell Correlation
Spatial Frequency (1/Å)
Corrected
Unmasked
Masked
FSC=0.143
f
g
K76
K89
H207
K78
Q43
V48
Q174
R46
H2A
H2B
Cse4
H4
h
72 bp
63 bp
Dyad
2
65
74
83
137
CEN3
TCGG
TTTATTTTTAAAAAATAAA
AAGCT
palindromic
65
83
i
91A
92G
51A
52A
39T
38T
37T
36T
109T
110C
111C
112G
j
74
137
k
Cse4CEN3
Cse4601

Supplementary Fig. 1. Cryo-EM structure of the native CEN3 CENP-A^{Cse4} nucleosome

a. Cryo-EM raw micrograph.

b. 2D class averages at different views.

c. Orientation distribution of the consensus refinement.

d. Data processing workflow of the scFv$_2$-CEN3 CENP-A^{Cse4} nucleosome.

e. Fourier shell correlation (FSC) curves.

f. Local resolution map.

g. Density maps and structural models for representative regions.

h. Illustration of the uneven length of the DNA between the dyad and the two ends, and the palindromic feature of the dyad region. The latter allows the identification of the dyad from the density map.

i. Unpolished density maps showing that only one orientation of the DNA (blue) fits the density well, whereas the opposite orientation of the DNA (magenta) leads to poor fitting of the density.

j. Illustration of models showing that the length of the shorter arm in the CEN3 DNA cannot account for all the density observed for the DNA at one end, excluding this DNA orientation (magenta).

k. Structural alignment of cryo-EM structures of CENP-A^{Cse4} nucleosomes containing 601 (cyan, PDB ID: 6UPH) and CEN3 DNA respectively, showing additional 4 bp DNA (black circle) in well-structured region for the nucleosome with 601 DNA. RMSDs of core histones: 1.4 for whole atoms, 0.9 for backbone.

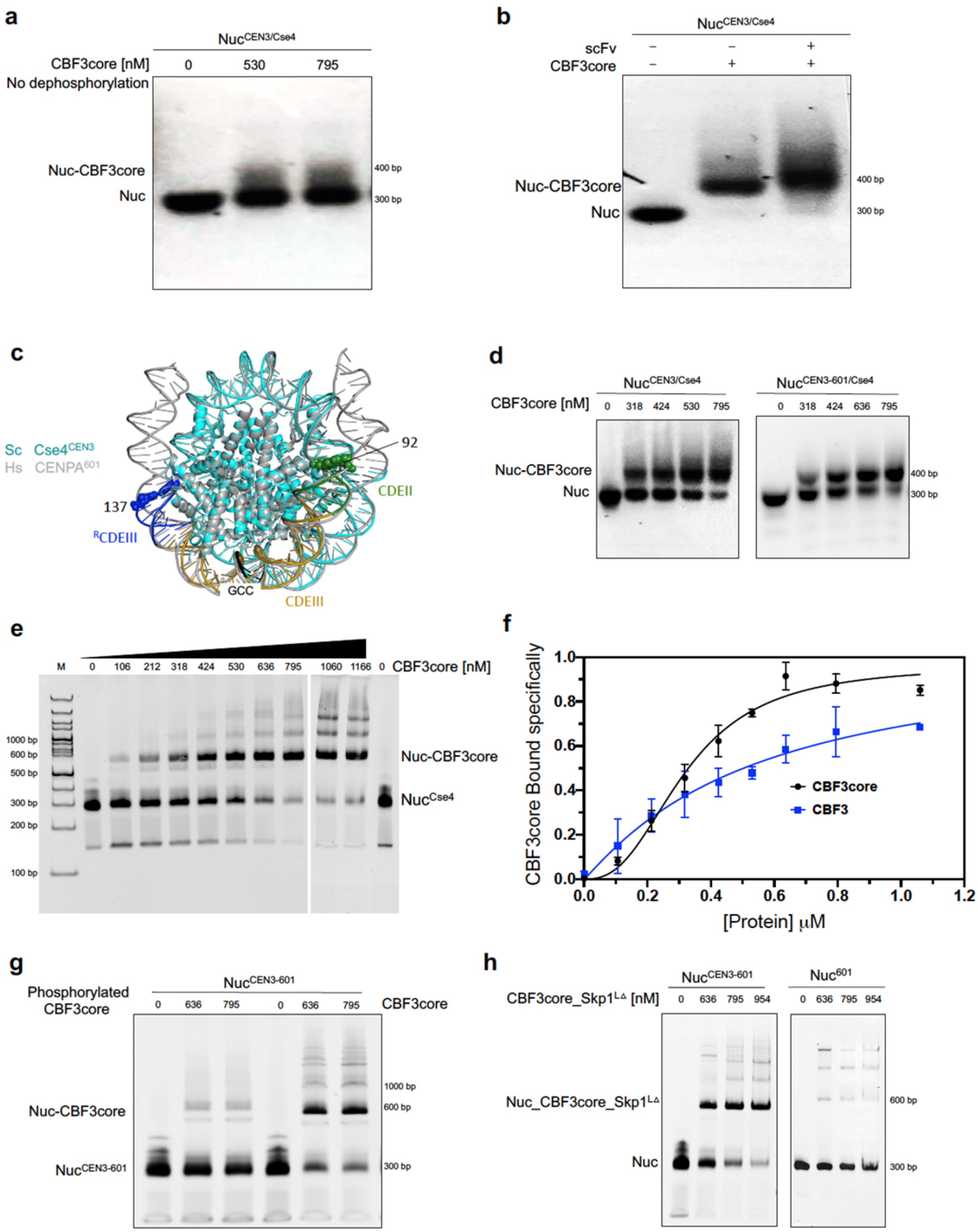
a
NucCEN3/Cse4
CBF3core [nM] 0 530 795
No dephosphorylation
Nuc-CBF3core
Nuc
400 bp
300 bp
b
NucCEN3/Cse4
scFv
CBF3core
Nuc-CBF3core
Nuc
400 bp
300 bp
c
Sc Cse4CEN3
Hs CENPA601
92
137
CDEII
RCDEIII
GCC
CDEIII
d
NucCEN3/Cse4
NucCEN3-601/Cse4
CBF3core [nM] 0 318 424 530 795
0 318 424 636 795
Nuc-CBF3core
Nuc
400 bp
300 bp
e
M 0 106 212 318 424 530 636 795 1060 1166 0 CBF3core [nM]
1000 bp
600 bp
500 bp
300 bp
200 bp
100 bp
Nuc-CBF3core
NucCse4
f
CBF3core Bound specifically
[Protein] μM
CBF3core
CBF3
g
Phosphorylated CBF3core
NucCEN3-601
0 636 795 0 636 795
CBF3core
Nuc-CBF3core
NucCEN3-601
1000 bp
600 bp
300 bp
h
NucCEN3-601
Nuc601
CBF3core_Skp1LΔ [nM] 0 636 795 954 0 636 795 954
Nuc_CBF3core_Skp1LΔ
Nuc
600 bp
300 bp

Supplementary Fig. 2 Binding of CBF3core to various nucleosomes.

a. EMSA assay showing that CBF3core purified from budding yeast cells without dephosphorylation by lambda phosphatase protein (λ-PP) could not bind to the nucleosome specifically.

b. EMSA assay of binding of CBF3core to the CEN3 CENP-A^{Cse4} nucleosome. All samples were incubated at room temperature and run in the 1% agarose gel. Addition of scFv caused smear of the band of the nucleosome bound to CBF3 core.

c. Structural comparison of CEN3 Cse4 nucleosome (cyan) with 601 CENP-A nucleosome (PDB 6BUZ, grey) and the re-engineered CDEII (green) CDEIII (yellow) and CDEIII^{R} (blue) regions are highlighted.

d. CBF3core can bind to the CEN3 and CEN3-601 CENP-A^{Cse4} nucleosomes with similar affinity. All samples were incubated at room temperature and run in the 0.8% agarose gel.

e. EMSA assay showing that CBF3core shifts the CEN3-601 Cse4 nucleosome.

f. Specific binding of CBF3core (black) and CBF3 (blue) to the nucleosome. The plot shows the averaged values (the intensity ratio of the first band above the free nucleosome over the free nucleosome) from three independent experiments. The error bars are the standard deviation. K_D values for CBF3core and CBF3 bindings are 0.32 ± 0.06 μM and 0.55 ± 0.06 μM, respectively.

g. EMSA assay showing the λ -phosphatase dependency of the CBF3core complex for binding of the CEN3-601 nucleosomes.

h. CBF3core_Skp1$^{L\Delta}$ with a deletion of the N-terminal loop of Skp1 (residues 37–64), which is the site of phosphorylation, bound to the CEN3-601 nucleosome with similar affinity as the λ-phosphatase-treated CBF3core but did not bind to the 601 nucleosome. All samples were incubated at 4 ºC for 1 hour and run in 4 % Native PAGE gels.

Uncropped gel images with molecular marks are provided as a Source Data File.

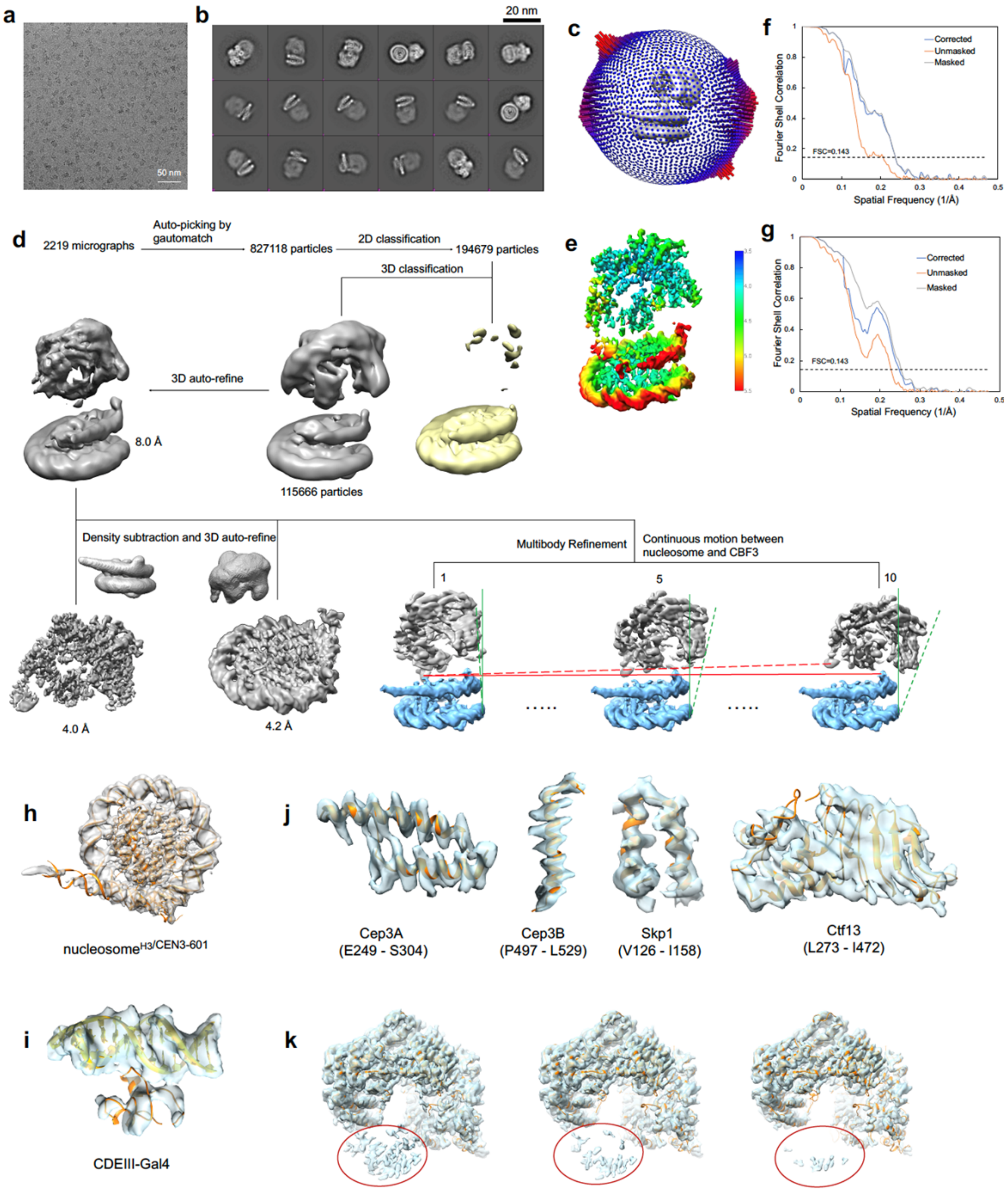

Supplementary Fig. 3 Structure of the CBF3core-CEN3-601 nucleosome

a. The raw micrograph.

b. 2D classes of different views.

c. Orientation distribution of the particles.

d. Data processing workflow.

e. The local resolution map.
f. The FSC curve of the nucleosome.
g. The FSC curve of the CBF3core after density subtraction.
h-j. Density maps and structural models of representative regions of Cenp3A, Cenp3B, Skp1, Ctf13, the nucleosome and interactions of CDEIII DNA and Gal4.
k. Illustration of the unassigned weak densities.

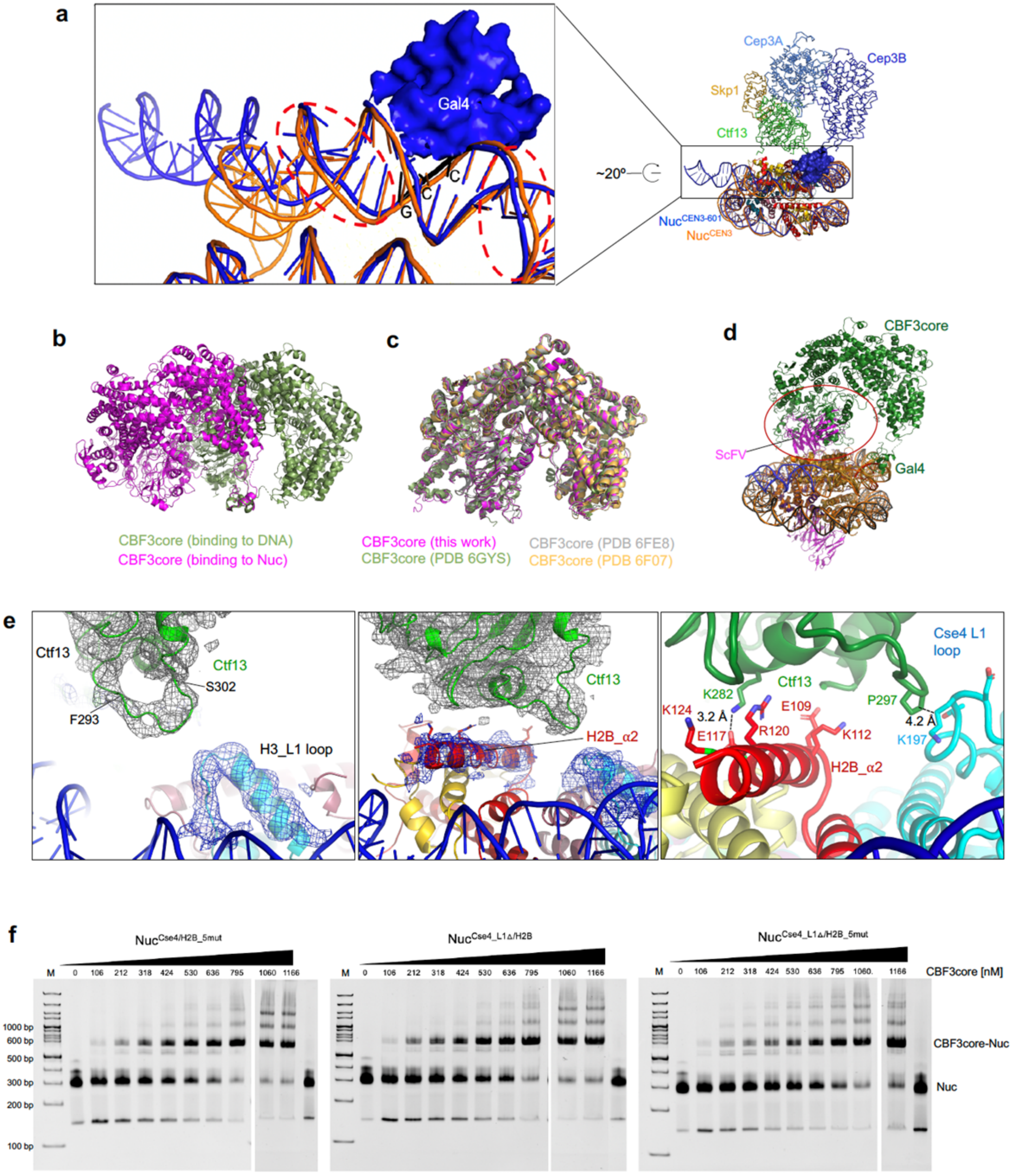

Supplementary Fig. 4 Interactions between CBF3core and the nucleosomes.

a. Alignment of the structures of the CBF3core-nucleosome complex and the free CEN3 nucleosome on the core histones. DNAs in the CBF3core-nucleosome complex and the CEN3 nucleosome are colored with blue and orange, respectively.

The Gal4 domain is shown in blue surface. The dashed ovals in red highlight the differences in the DNA conformations.

b. Alignment of the Gal4 domains in the CBF3core bound to the free DNA (PDB 6YGS, green) and the nucleosome (magenta).

c. Alignment of the major domains of CBF3cores (PDB 6FE8, grey; PDB 6F07, yellow; PDB 6YGS, green; CBF3core bound to nucleosome, magenta) showing the major domains of the CBF3core have a rigid structure.

d. Alignment of the structures of the nucleosomes bound to CBF3core and scFv showing clash between CBF3core and scFv.

e. Local density maps at the interface between Ctf13 and core histones (left and middle) and potential formation of salt bridge between Ctf13 K282 and H2B E117 and hydrophobic interactions between Cft13 P297 and Cse4 K1987 in the CBF3core-Cse4 nucleosome model (right).

f. EMSA assay of binding of the CEN3-601 nucleosomes with H2B mutations and Cse4 L1 loop deletions to CBF3core. All samples were incubated at 4 ºC for 1 hour and run in 4 % Native PAGE gels. Uncropped gel images are provided as a Source Data File.

Supplementary Table 1 | Cryo-EM data collection, refinement and validation statistics

	Nucleosome-scFv (EMDB-22696) (PDB 7K78)	Nucleosome-CBF3core	
		Nucleosome (EMDB-22698) (PDB 7K7G)	CBF3core (EMDB-22697) (PDB 7K79)
**Data collection and processing**			
Magnification	130,000	130,000	130,000
Voltage (kV)	300	300	300
Electron exposure (e–/Å^2)	71	71	71
Defocus range (μm)	-1.0 to -2.0	-1.0 to -2.0	-1.0 to -2.0
Pixel size (Å)	1.06	1.06	1.06
Symmetry imposed	C1	C1	C1
Initial particle images (no.)	342,749	827,118	827,118
Final particle images (no.)	143,164	115,666	115,666
Map resolution (Å)	3.1	4.2	4.0
FSC threshold	0.143	0.143	0.143
**Refinement**			
Map sharpening *B* factor (Å^2)	-82	-100	-100
Model composition			
Non-hydrogen atoms	14,125	11,098	13,274
Protein residues	1,198	766	1,601
Nucleotide	232	246	-
B factors (Å^2)			
Protein	21	206	145
Nucleotide	72	310	-
R.m.s. deviations			
Bond lengths (Å)	0.004	0.007	0.009
Bond angles (°)	0.685	1.023	1.804
Validation			
MolProbity score	1.59	1.71	2.11
Clashscore	8.36	7.76	11.25
Poor rotamers (%)	0.20	0.00	0.13
Ramachandran plot			
Favored (%)	97.27	95.86	90.37
Allowed (%)	2.73	4.14	9.63
Disallowed (%)	0.00	0.00	0.00

Supplementary Table 2 | A complete list of all primers

CEN3-601_F	ATCGAGAATCCCGGTG
CEN3-601_R	ATCGGATGATTTCTTACTATTTC
CSE4_L1_TKD_deletion_F	GAAGTTACTGATGAGTTCACCCAGGACCTGA GATGGCAGAGTATG
CSE4_L1_TKD_deletion_R	CATACTCTGCCATCTCAGGTCCTGGGTGAACT CATCAGTAACTTC
Y_H2B_E117A/R120A/K124A_F	CATGCTGTCTCTGCTGGTACTGCTGCTGTTAC CGCTTACTCTTCCTCTACTCAAGC
Y_H2B_E117A/R120A/K124A_R	GGAAGAGTAAGCGGTAACAGCAGCAGTACCA GCAGAGACAGCATGCTTAGCCAA
Y_H2B_E109A/K112A_F	TTGATCTTACCAGGTGCGTTGGCTGCGCATG CTGTCTCTGCTGGTACTGCTGCTGTTAC
Y_H2B_E109A/K112A_R	AGCAGAGACAGCATGCGCAGCCAACGCACCT GGTAAGATCAATCTAACAGCGGTTTG
