# Supplementary material for: Structural and dynamic mechanisms of CBF3-guided centromeric nucleosome formation
Source: Nat Commun. 2021 Mar 19;12:1763. doi: 10.1038/s41467-021-21985-9 (PMC7979930; doi:10.1038/s41467-021-21985-9)
Supplement: Supplementary file 3 — Description of Additional Supplementary Files [file 41467_2021_21985_MOESM3_ESM.pdf]

Description of Additional Supplementary Files

File name: Supplementary Movie 1

Description: Dynamic motions of main CBF3core region relative to the nucleosome
